# RETRACTION: The Diagnostic Value and Associated Molecular Mechanism Study for Fibroblast‐Related Mitochondrial Genes on Keloid

**DOI:** 10.1111/srt.70188

**Published:** 2025-06-05

**RETRACTION**: T. Wei, and Z. Xu, “The Diagnostic Value and Associated Molecular Mechanism Study for Fibroblast‐Related Mitochondrial Genes on Keloid,” *Skin Research and Technology* 30, no. 9 (2024): e70024, https://doi.org/10.1111/srt.70024.

The above article, published online on 2 September 2024 in Wiley Online Library (wileyonlinelibrary.com), has been retracted by agreement between *Skin Research and Technology*; and John Wiley & Sons Ltd. The article has been accepted for publication following an inadequate peer review process. Following publication of the article, several flaws and inconsistencies between results presented and methodology described were found. Furthermore, several claims lack sufficient support from the literature. Accordingly, the article is retracted as the editors have lost trust in the reliability of the results presented. The authors have been informed of the decision of retraction and disagree with it.

